# Sustained Autism Outcomes Eight Years After Early Intensive Behavioral Intervention in a Conflict-Affected Low-Resource Setting: A Longitudinal Follow-Up Study

**DOI:** 10.1007/s10802-026-01438-x

**Published:** 2026-02-26

**Authors:** Wissam Mounzer

**Affiliations:** 1 Department of Psychology, Damascus University, Damascus, Syria; 2 Department of Special Education, Stockholm University, Stockholm, 106 91 Sweden

**Keywords:** Autism spectrum disorder, Early intensive behavioral intervention, Longitudinal outcomes, Adaptive functioning, Low-resource settings, Syria

## Abstract

This study examined the long-term outcomes of the Future Centre’s Early Intensive Behavioural Intervention (FC-EIBI) for children with autism spectrum disorder (ASD) in a conflict-affected, low-resource setting in Syria. Sixty-six participants were assessed at baseline (2008), post-treatment (2010), early follow-up (2013), and long-term follow-up (2019) using the Childhood Autism Rating Scale (CARS), the Autism Behaviour Checklist–Arabic (ABC), and the Adaptive Behaviour Scale–Arabic (ABS-Arabic). Longitudinal change was examined using nonparametric repeated-measures analyses, with additional analyses of subdomains across six ABS-Arabic domains. Significant improvements were observed across all measures from baseline to post-treatment and early follow-up (all ps < 0.001), indicating substantial reductions in autism symptom severity and marked gains in adaptive functioning. By 2019, small but significant attenuation of earlier gains was detected; however, outcomes remained significantly improved relative to baseline. Improvements in adaptive functioning were most pronounced between 2008 and 2013, particularly in Social Interaction and Communication/Language, whereas later declines were most evident in Communication/Language and Personal–Emotional Adaptation. Overall, FC-EIBI was associated with considerable and durable developmental benefits over more than a decade, despite prolonged sociopolitical instability and service disruptions. These findings underscore the feasibility of sustaining meaningful developmental outcomes for children with ASD in conflict-affected, low-resource contexts.

**Supplementary Information:**

The online version contains supplementary material available at 10.1007/s10802-026-01438-x.

## Introduction

Autism spectrum disorder (ASD) is a lifelong neurodevelopmental condition characterized by differences in social communication and interaction alongside restricted interests and repetitive behaviors, with onset in early childhood (American Psychiatric Association, 2013). Over the last three decades, a converging empirical literature has shown that early, structured, and individualized behavioral intervention can yield substantial developmental gains for many children on the spectrum. Early Intensive Behavioral Intervention (EIBI), grounded in Applied Behavior Analysis (ABA), is among the most extensively studied of these approaches, with repeated demonstrations of improvement in language, cognitive ability, and everyday functioning when treatment is delivered intensively and with high fidelity (Ben-Itzchak & Zachor, 2007; Eldevik et al., 2009; Eikeseth et al., 2012; Estes et al., 2015; Lovaas, 1987; Peters-Scheffer et al., 2011; Reichow et al., 2018; Warren et al., 2011). While this evidence base has shaped international recommendations for early care, it has also sharpened a central question for developmental science and clinical practice: to what extent do early gains translate into adaptive functioning later in life, and under what conditions are those gains sustained?

A growing body of longitudinal work positions adaptive functioning—the capacity to apply skills in real-world contexts—as a critical endpoint for autism interventions and a more proximal predictor of adult independence than IQ alone (Bal et al., 2021; Szatmari et al., 2019). In a recent longitudinal study following adolescents with and without ASD into emerging adulthood, Fossum et al. (2025) reported that higher adaptive skills earlier in development, lower concurrent symptom load, and stronger contextual supports (e.g., sustained parental involvement, access to services) predicted better functional outcomes several years later. In contrast, IQ showed weaker associations when adaptive skills were modelled directly. This work underscores two central points of the present study. First, adaptive functioning is not merely a downstream correlation of cognitive ability; it is a malleable, context-sensitive developmental construct that integrates social participation, daily living, and practical problem-solving. Second, the durability of early improvements depends on the continuity of opportunities to practice and generalize skills across the life course. This continuity is often assumed in well-resourced settings but is frequently disrupted elsewhere.

These insights complement and reframe earlier debates about the long-term maintenance of EIBI effects. Classic follow-ups reported sustained benefits years after treatment ended (McEachin et al., 1993; Perry et al., 2017; Smith et al., 2021), whereas other reviews and studies emphasized heterogeneity and partial attenuation over time (Howlin et al., 2009; Matson & Konst, 2013; Tonge et al., 2014). A systematic review of long-term outcomes highlighted a shortage of genuine multi-year follow-ups, particularly those extending beyond five years, and called for designs that span key developmental transitions (Steinhausen et al., 2016). Taken together with Fossum et al. (2025), this literature suggests that the question is not simply whether early gains “last,” but which early experiences and later contexts support the consolidation of adaptive skills as adolescents move toward adult roles.

At the same time, the field faces challenges with cultural generalizability. Much of what is known about EIBI and long-term outcomes comes from WEIRD (Western, educated, industrialized, rich, democratic) contexts (Muthukrishna et al., 2020), where relatively stable infrastructures and professional resources can scaffold ongoing skill practice. Scholars in behavior analysis and special education have argued that cultural humility, social validity, and contextual fit are prerequisites for impactful intervention (Sugai et al., 2012; Miller et al., 2019). In the Arab region, empirical reports document growing interest in behavioral approaches but also substantial variability in training, supervision, and system capacity (Al-Hemoud & Al-Asfoor, 2006; Eapen et al., 2007; Hussein & Taha, 2013; Kelly et al., 2016; Sartawi, 1999). These realities raise an unresolved question that is foundational for a global science of autism intervention: Can early behavioral gains be sustained where services are scarce and life conditions are unstable?

Syria provides a compelling case in point of underrepresentation. Prior to 2008, structured ABA-based services aligned with international EIBI standards were largely unavailable. The establishment of the Future Center EIBI (FC-EIBI) program marked a pivotal shift by introducing a comprehensive, two-year model that combines approximately 25 h per week of individualized center-based instruction with systematic parent coaching and home practice for young children aged 1–6 years (Mounzer & Stenhoff, 2022). The original evaluation, using the Childhood Autism Rating Scale (CARS) (Schopler et al., 1980), the Autism Behavior Checklist (ABC; Volkmar et al., 1988; Arabic version by Ghazal, 2007), and the Adaptive Behavior Scale–Arabic (ABS-Arabic; Al-Kilani & Al-Batesh, 1981), demonstrated significant reductions in autism symptom severity and large gains in adaptive functioning across social-communication, self-care, motor, and pre-academic domains. These findings established both the feasibility and promise of EIBI in a low-resource, Arabic-speaking context.

The outbreak of civil war transformed the developmental ecology of participating families. Schooling, healthcare, and rehabilitation infrastructures were disrupted; many families experienced displacement, economic hardship, and loss of community support. Despite these adversities, a three-year follow-up of the same cohort (Mounzer et al., 2023) reassessed 66 of the original 67 children and found continued reductions in symptom severity and further improvements in adaptive behavior, with only modest regression in social-communication subdomains. Notably, the frequency of structured learning trials—an index of sustained practice—remained positively associated with adaptive outcomes and negatively associated with symptom severity, suggesting that parent-mediated opportunities to rehearse skills helped preserve gains even after formal services ended. These results align closely with Fossum et al. (2025), who reframed adaptive functioning as a dynamic construct continually shaped by access to practice, social participation, and contextual supports across adolescence.

Framed within this developmental-ecological perspective, the Syrian findings raise a critical question for theory and policy: Can early intensive intervention produce adaptive competencies robust enough to withstand years of environmental disruption? Western longitudinal studies have shown that gains persist when educational supports and parental reinforcement continue (Estes et al., 2015; Smith et al., 2021), but the mechanisms underlying maintenance are intertwined with the stability of those systems. In contrast, families in conflict-affected settings often shoulder the primary responsibility for sustaining skill use with minimal professional scaffolding. UNICEF (2019) has documented the scale of disruption to children’s education and services in Syria, underscoring the rarity of multi-wave longitudinal data in such contexts.

The present study advances this evidence base by reporting an eight-year follow-up of the FC-EIBI cohort, extending the observation window to four waves across eleven years (2008 baseline; 2010 post-treatment; 2013 early follow-up; 2019 long-term follow-up) and employing the same standardized measures used at intake. Consistent measurement allows us to model within-person changes over extended periods and to characterize both consolidation and attenuation of earlier gains as children transition into adolescence. Conceptually, our design is guided by the framework articulated by Fossum et al. (2025) and related longitudinal work (Bal et al., 2021; Szatmari et al., 2019): adaptive functioning is treated as a primary outcome of enduring clinical significance rather than a secondary correlation of cognition. In line with that framework, we interpret stability or regression of adaptive behavior not only as an index of treatment durability but also as a signal of the opportunities—and constraints—afforded by children’s environments over time.

This perspective has several implications. First, maintaining adaptive behavior is effortful: without continued practice in natural contexts, even well-learned skills can degrade. For families in low-resource, conflict-affected settings, creating the conditions for practice (e.g., consistent routines, peer interaction, access to school) often requires substantial improvisation and resilience. Second, because adaptive functioning encompasses daily living, social participation, and communication, it is susceptible to disruptions in schooling and community life—domains that are heavily affected by conflict. Third, when treatment benefits do persist under these conditions, they provide high-value evidence for the ecological validity of early behavioral approaches and for the central role of parental engagement as an enduring mechanism of change (Epstein et al., 2002; Granger et al., 2012; Sugai et al., 2012; Miller et al., 2019).

Against this backdrop, our aims are deliberately conservative and empirically focused. We evaluate whether the substantial improvements observed immediately after FC-EIBI and at the 2013 follow-up were sustained at the 2019 assessment across three domains: autism symptom severity (CARS), maladaptive behaviors (ABC), and adaptive functioning (ABS-Arabic). We also characterize the patterning of change—whether gains plateau, continue, or partially regress—across subdomains of adaptive behavior that are theoretically vulnerable to environmental disruption (e.g., social communication; personal–emotional adaptation) versus those that may be more resilient (e.g., self-care; motor skills). By situating our findings within a longitudinal framework that centers adaptive functioning (Fossum et al., 2025; Bal et al., 2021; Szatmari et al., 2019), we aim to inform a global conversation about how early intervention interacts with context to shape life-course trajectories.

In sum, this eight-year follow-up contributes three advances. First, it provides one of the longest longitudinal datasets on EIBI outcomes available and the only one— to our knowledge—conducted in a conflict-affected, low-resource setting using consistent instruments across four waves. Second, by foregrounding adaptive functioning as the core endpoint and interpreting change through a developmental-ecological lens, it aligns with and extends contemporary longitudinal models of life-course outcomes in ASD (Fossum et al., 2025). Third, the study has immediate translational value: it highlights the feasibility of culturally adapted, parent-mediated behavioral programs and points to community-based strategies that may help consolidate early gains where formal services are intermittent. These contributions are essential for an equitable science of autism intervention—one that recognizes that the durability of early gains depends not only on what is learned in therapy, but also on where and how children live, practice, and participate as they grow.

Importantly, the present study is not designed to establish the causal efficacy of EIBI relative to alternative interventions or typical development. Rather, it documents within-individual developmental trajectories over an extended period following a well-specified early intervention in a conflict-affected, low-resource context. Accordingly, the analytic focus is on the durability, attenuation, and domain-specific patterning of change, rather than on causal attribution.

## Methods

### Setting and Participants

Families were contacted by phone calls and e-mail between May and October 2019 to participate in this long-term follow-up of the *Future Center–Early Intensive Behavioral Intervention* (FC-EIBI) program. Sixty-six of the original 67 participants with autism spectrum disorder (ASD) who were enrolled in the FC-EIBI program between 2008 and 2010 (Mounzer & Stenhoff, 2022) and re-evaluated in 2013 (Mounzer et al., 2023). Data collection occurred in 2019; for clarity, we refer to this wave as the 2019 follow-up. Only one participant from the original cohort was deceased.

All participants had previously received a formal diagnosis of ASD based on the *Diagnostic and Statistical Manual of Mental Disorders (DSM-IV-TR; American Psychiatric Association*,* APA*,* 2000)* and the *Wechsler Intelligence Scale for Children–IV* administered at intake in 2008. The diagnosis was confirmed by the psychologist using DSM criteria. Diagnostic confirmation was based on the DSM-IV-TR, which was the standard at the time of assessment (2008). DSM-5 criteria (APA, 2013) were adopted only for interpretive consistency in later analyses. The Arabic version of the Wechsler Intelligence Scale for Children—Fourth Edition (WISC-IV; Wechsler, 2003) was administered individually by licensed psychologists trained in standardized administration. All testers completed a two-day reliability workshop, and inter-rater reliability (intra-class correlation coefficient) exceeded 0.90 across subscores. The mean IQ was 61 (range = 39–83), consistent with the earlier assessments. Participants had completed two consecutive years of EIBI at the Future Center and had been out of the program for approximately eight years at the time of the current follow-up.

At the time of intake (2008), diagnostic confirmation was based on DSM-IV-TR criteria applied by licensed psychologists with extensive clinical experience in ASD. Gold-standard research instruments such as the Autism Diagnostic Observation Schedule (ADOS) and Autism Diagnostic Interview–Revised (ADI-R) were not available in Arabic nor routinely implemented in clinical practice in Syria during that period. As such, diagnoses reflect real-world clinical procedures typical of low-resource and conflict-affected settings. While this limits comparability with studies using the ADOS/ADI-R, it enhances ecological validity and accurately reflects the service conditions under which the intervention was delivered.

As a reassessment, the participants’ ages ranged from 13 to 18 years (*M* = 15.3, *SD* = 1.8). 36% (*n* = 24) were aged 13–14 years, 33% (*n* = 22) were aged 15–16 years, and 31% (*n* = 20) were aged 17 years or older. The sample included 38 boys (58.2%) and 28 girls (41.8%).

80% of the participants (*n* = 53) were still residing in Syria and were re-evaluated at the Future Center in Damascus. The remaining 20% (*n* = 13) had migrated to Europe because of the civil war but returned to Syria temporarily in 2019 to participate in the follow-up assessment. These families were residing in Germany (*n* = 6), Spain (*n* = 3), Norway (*n* = 2), and Sweden (*n* = 2).

The social and demographic characteristics of the families remained similar to those reported in the 2013 study: 29% of families had a low educational level, 28% reported low income, 24% were divorced, and 19% were single-parent households (Mounzer et al., 2023) Many families (*n* = 37) had been internally displaced during the years following the conflict, though most had returned to their original residences by the time of this follow-up. Socioeconomic data were collected using a structured parental questionnaire that covered education, employment, and household income. This instrument was adapted from the UNICEF Child and Family Survey (2018) and demonstrated high test–retest reliability (*r* =.89) in pilot use with Syrian families.

Approximately 46% of participants continued to receive pharmacological treatment (e.g., risperidone, atomoxetine, or valproate), and around one-third received occasional speech, language, or occupational therapy. As in previous studies, medication and external therapies were not controlled by the analyses.

All 2019 assessments were conducted by three licensed Syrian psychologists who had no prior relationship with the participants, their families, or the Future Center. The assessors were independent of the original program staff and blind to participants’ earlier scores. The author supervised and verified all data collection procedures to ensure methodological consistency with the earlier assessment waves.

At the 2019 follow-up, participants were re-contacted through telephone directories, local NGOs, and school networks. Families who had relocated were reached via WhatsApp and community mediators. This multi-channel strategy minimized attrition; only one participant could not be located. Attrition analyses were not performed because only one participant was lost to follow-up, resulting in 98.5% retention—an unusually high rate for longitudinal studies in conflict settings.

## Procedures

### FC-EIBI Program

The following summary provides operational details of the 2008–2010 intervention, drawn from contemporaneous program documentation (Mounzer & Stenhoff, 2022). The *Future Center–Early Intensive Behavioral Intervention* (FC-EIBI) program was established at the *Future Center for Children with Special Needs* in Damascus to provide structured behavioral treatment for young children diagnosed with autism spectrum disorder (ASD). The program targeted children aged 1 to 6 and was implemented between 2008 and 2010. Intervention fidelity during 2008–2010 was documented through weekly supervision checklists and monthly video reviews; therapist adherence to individualized programs averaged 91% (*SD =* 4.2), confirming high implementation integrity.

The FC-EIBI program was designed to teach parents to act as active co-therapists for their children and to facilitate their children’s success across home, school, and community environments. The intervention model was grounded in the principles of *Applied Behavior Analysis* (ABA) and incorporated the following core components: (a) theoretical instruction for parents, (b) practical workshops and guided practice sessions, (c) ongoing documentation and assessment of progress, (d) consistent communication and feedback between staff and families, and (e) systematic implementation of individualized teaching plans (Mounzer & Stenhoff, 2022).

Each child receives approximately 25 h of one-to-one EIBI per week over 2 years. Children attended the center daily from 8:30 a.m. to 1:30 p.m., participating in 10 structured 30-minute teaching sessions. Parents received 5 h of weekly coaching and were instructed to conduct two additional home-based practice sessions per week.

The instructional curriculum focused on six major developmental domains: social interaction, communication and language, self-care, gross and fine motor skills, emotional and behavioral regulation, and pre-academic skills. On average, children completed 39 learning trials per week (range 36–45) and achieved mastery in approximately 52 target skills per year.

Behavioral teaching methods included discrete-trial training, prompting and fading, reinforcement, shaping, and generalization of acquired skills across multiple settings. Each child’s progress was documented daily and reviewed during weekly therapist meetings. The most frequently targeted areas of development were communication and social interaction. Trials were adapted according to each child’s acquisition rate (*M* = 1 week per skill).

Trained therapists delivered all instruction under continuous supervision from senior program staff. Parent participation was a central element of the model, and parents received written feedback and structured homework tasks after each coaching session. The FC-EIBI program emphasized the consistency of behavioral strategies between home and center environments to maximize skill generalization.

###  Design

We followed up on the initial study (Mounzer & Stenhoff, 2022) and evaluated the long-term effects of the FC-EIBI program on 66 participants using a longitudinal quasi-experimental design. Changes in participants’ scores on the ABC, CARS, and ABS-Arabic were analyzed across four time points (2008, 2010, 2013, and 2019). Assessments were conducted at four time points approximately 24 months (2008–2010), 36 months (2010–2013), and 72 months (2013–2019) apart, providing an 11-year observation window. The quasi-experimental longitudinal design was selected because ethical and contextual constraints in a conflict-affected country precluded randomization or the use of a control group. All assessments were conducted by independent psychologists who were not affiliated with the Future Center. To reduce assessment bias, evaluators were blind to participants’ prior test scores and to the chronological order of assessment waves. Reliability across assessors was examined on 20% of overlapping cases, yielding intraclass correlation coefficients *(ICC[2*,*1])* between 0.92 and 0.95 for total scale scores.

### Measures

The ABC, CARS, and ABS-Arabic scales were selected for this study because they were used in the initial study (Mounzer & Stenhoff, 2022) and demonstrate documented internal consistency, test–retest reliability, and prior validation in Arabic-speaking populations. In the initial study, we computed a high level of internal consistency across the scales (*α*_ABC_ = 0.95; *α*_CARS_ = 0.98; *α*_ABS_ = 0.87). Additionally, there was high test-retest reliability across the scales (*r*_ABC_=0.96, *r*_CARS_=0.91, *r*_ABS_=0.87, *p*<.001). These reliability estimates are consistent with previously reported validation studies of the Arabic versions of these instruments, including Ghazal (2007) for the ABC, Al-Shammari and Al-Sartawi (2002) for the CARS, and Al-Kilani and Al-Batesh (1981) for the ABS-Arabic, all of which demonstrated satisfactory internal consistency and cultural validity.

#### Cross-cultural Applicability of Measures

Formal tests of configural, metric, and scalar invariance were not feasible because there were no original normative datasets and item-level calibration parameters for the source populations of these instruments. Consequently, the present analyses do not assume cross-cultural score equivalence with Western norms. Instead, all interpretations are restricted to within-individual change over time using identical instruments administered longitudinally. This approach aligns with recommendations in cross-cultural methodology, where intra-individual trajectories may be meaningfully interpreted even in the absence of full measurement invariance, provided that instruments are applied consistently and demonstrate acceptable internal reliability within the target population (Chen, 2007; Hui & Triandis, 1985; Van de Vijver & Leung, 1997).

The Arabic version of the ABC, developed by Ghazal (2007), consists of 57 items grouped into five subscales: sensory (9 items), relating (12 items), body and object use (13 items), language (12 items), and social/self-help skills (11 items). Items are rated on a four-point scale from 1 (appropriate behavior) to 4 (severely atypical behavior). Completion typically takes 10–20 min and can be done by parents or teachers who have known the child for at least three to six weeks. Total scores above 67 indicate the presence of ASD (Volkmar et al., 1988); higher totals reflect greater symptom severity. Internal consistency in the current sample was *α = 0.95*, confirming excellent reliability.

The CARS (Al-Shammari & Sartawi, 2002; Schopler et al., 1980) comprises 15 items assessing behaviors across 14 domains and one global impression of autism severity. The domains include visual response, relating to people, emotional response, body and object use, adaptation to change, activity level, listening response, intellectual consistency, perceptual response, fear or anxiety, verbal and nonverbal communication, and imitative behavior. Each item is scored from 1 (age-appropriate) to 4 (severely deviant). Scores below 30 indicate non-ASD; scores 30–37 indicate mild to moderate ASD; and scores ≥ 38 reflect severe ASD. The Arabic adaptation validated by Al-Shammari and Al-Sartawi (2002) reported internal consistency (*α* = 0.79) and inter-rater reliability above 0.90; reliability in the current sample (α *=.*98) was consistent with these findings.

The ABS-Arabic (Al-Kilani & Al-Batesh, 1981) was developed and standardised for Arabic-speaking populations, with items derived from culturally normative expectations regarding daily living, social participation, and communication. As shown in the original manual (Al-Kilani & Al-Batesh, 1981), domains emphasize functional behaviors observable in family and community contexts, enhancing cultural relevance for the present sample. It includes 96 items distributed across six major domains: communication and language, social interaction, self-care, gross and fine motor skills, personal and emotional adaptation, and cognitive skills. Each domain has two subdomains, each with eight items. Items are rated on a four-point scale from 1 (age-appropriate) to 4 (severely deviant). The total score reflects overall adaptive functioning; higher scores indicate stronger adaptive abilities. Because local standardized norms were unavailable, raw domain and total scores were used in analyses; internal consistency in the current study was *α* = 0.87.

### Fidelity Assessment

Separate fidelity procedures were implemented for both intervention delivery (2008–2010) and the 2019 assessment phase. The author supervised and monitored the fidelity of the assessment procedures during the 2019 data collection. Fidelity data were collected for 40% of all assessment sessions, including both in-person evaluations and online interview sessions. The same fidelity checklist used in previous assessments was employed, adapted to include both standardized testing and interview procedures.

The checklist included the following steps: (a) confirming the assessor’s preparation and adherence to standardized administration protocols; (b) providing positive feedback regarding performance in the previous session; (c) identifying areas requiring improvement; (d) providing immediate corrective feedback for both accurate and inaccurate steps; (e) ensuring participant comfort and compliance with ethical standards; (f) asking the assessor to clarify any uncertainties; and (g) concluding the session with a summary and positive statement.

The face validity of the fidelity checklist was confirmed through independent review by two senior experts in autism assessment and ABA methodology. Fidelity scores were calculated as the percentage of steps completed correctly and averaged 98.4% across all observed sessions. Inter-observer agreement between the author and the external reviewers was 95.7%, indicating high procedural reliability.

### Data Analysis

All statistical analyses were conducted using *IBM SPSS Statistics* version 29.0. Because distributions were non-normal, descriptive statistics are presented as medians (IQR) in the text for omnibus outcomes and as means *(SD)* in tables for subdomain descriptives; inferential tests used the Friedman and Wilcoxon procedures with four assessment points (2008, 2010, 2013, and 2019) as the within-subjects factor, and Bonferroni-adjusted Wilcoxon signed-rank comparisons quantified longitudinal change. Subdomain analyses were conducted for six ABS-Arabic domains. Effect sizes (*r* and *Kendall’s W*) were calculated following the recommendations of Rosenthal (1991) and Tomczak and Tomczak (2014), with *r = |Z|/√N* for Wilcoxon signed-rank tests and *W = χ²/[N × (k − 1)]* for Friedman tests. Interpretation of r followed Cohen’s (1988) benchmarks: *0.10 = small*,* 0.30 = medium*,* 0.50 = large.* Missing data were minimal (< 2%) and handled via pairwise deletion; analyses were performed on all available cases.

### Ethical Approval

All study procedures adhered to the *Code of Ethics of the World Medical Association (Declaration of Helsinki)* and its subsequent amendments. Ethical approval for the current follow-up was granted by the Ethics Committee of the Department of Psychology at Damascus University, in accordance with national and international regulations. Due to the first author’s current affiliation with Stockholm University, a supplementary ethics application was submitted and approved by the Swedish Ethical Review Authority (approval number 2022-01262-01).

Written informed consent was obtained from all participants and their parents or legal guardians before participation. Families were fully informed of the study’s purpose, procedures, and confidentiality policy. Participation was voluntary, and parents could withdraw their children from the study at any time without penalty. Families were also allowed to review their own data and decide which information to include in the final analysis. All identifying data were anonymized and stored securely in compliance with data protection regulations. To ensure participant safety during wartime, assessments were scheduled in secure facilities within the Future Center, and all files were encrypted and stored offline. In addition, assent was sought from participating children when developmentally appropriate, with procedures adapted to the child’s communicative abilities.

## Results

We examined changes in CARS, ABC, and ABS-Arabic across four assessments (2008, 2010, 2013, 2019). Given non-normality, Friedman tests were used for omnibus testing, and Bonferroni-adjusted Wilcoxon tests were used for pairwise comparisons.

### CARS, ABC, and ABS-Arabic Outcomes

There was a significant main effect of the time on participants’ CARS, ABC, and ABS-Arabic scores as revealed by Friedman tests, *χ²*(3) = 184.41, *p <*.001 for CARS; *χ²*(3) = 177.85, *p <*.001 for ABC; and *χ²*(3) = 162.39, *p <*.001 for ABS-Arabic (Table 1). These findings indicate substantial overall changes across the four assessment points (2008, 2010, 2013, 2019). Bonferroni-adjusted Wilcoxon signed-rank comparisons showed significant improvements between pre- and post-test scores for all three measures. CARS Scores showed marked improvement over time, reflected by sustained reductions in symptom severity from 2008 (*Mdn* = 36) to 2010 (*Mdn* = 33; *Z* = − 7.18, *p* <.001, *r* =.89) and continued to 2013 (*Mdn* = 31; *Z* = − 6.91, *p* <.001, *r* =.85). A statistically significant improvement with a large nonparametric effect size was observed from 2013 to 2019 (*Z* = − 6.75, *p* <.001, *r* =.83), indicating a regression (Table 1). However, 2019 scores remained substantially lower than baseline levels (*p* <.001). Hereafter, the term “regression” is used in the results section descriptively to denote partial loss of previously acquired gains rather than developmental reversal.

**Table 1:** Friedman and Bonferroni-adjusted Wilcoxon signed-rank tests for CARS, ABC, and ABS-Arabic scores (2008–2019)

Scale	*Friedman χ² (3,N= 66)*	*p*	Kendall’s W	Pairwise comparison	Z	*p*	*r*	Direction
CARS	184.41	< 0.001	0.93	2010–2008	−7.18	< 0.001	0.88	↓ Improvement
				2013–2008	−7.13	< 0.001	0.88	↓ Improvement
				2019–2008	−7.15	< 0.001	0.88	↓ Improvement
				2013–2010	−6.91	< 0.001	0.85	↓ Further gain
				2019–2013	−6.75	< 0.001	0.83	↑ Regression
ABC	177.85	< 0.001	0.90	2010–2008	−7.06	< 0.001	0.87	↓ Improvement
				2013–2008	−7.06	< 0.001	0.87	↓ Improvement
				2019–2008	−7.07	< 0.001	0.87	↓ Improvement
				2013–2010	−6.94	< 0.001	0.85	↓ Further gain
				2019–2013	−6.91	< 0.001	0.85	↑ Regression
ABS-Arabic	162.39	< 0.001	0.82	2010–2008	−7.06	< 0.001	0.87	↑ Improvement
				2013–2008	−7.06	< 0.001	0.87	↑ Improvement
				2019–2008	−7.06	< 0.001	0.87	↑ Improvement
				2013–2010	−5.83	< 0.001	0.72	↑ Continued gain
				2019–2013	−6.01	< 0.001	0.74	↓ Regression

*All Wilcoxon signed-rank tests were two-tailed and Bonferroni-adjusted (α = 0.05/6 = 0.0083). Z = standardized Wilcoxon test statistic; r = |Z|/√66 (√66 ≈ 8.12). ↑ = increase (improvement in adaptive behavior); ↓ = decrease (reduction in symptom severity). Effect size interpretation (* Cohen, 1988*):r =.10 small,0.30 medium, 0.50 large*

A similar pattern emerged for the ABC scores. Maladaptive behaviors decreased significantly from 2008 (*Mdn* = 119) to 2010 (*Mdn* = 96; *Z* = − 7.06, *p* <.001, *r* =.87) and further declined by 2013 (*Mdn* = 84; *Z* = − 6.94, *p* <.001, *r* =.86). By 2019, ABC scores (*Mdn* = 98) remained far below pre-intervention levels (*p* <.001) but were higher than at the 2013 follow-up (*Z* = − 6.91, *p* <.001, *r* =.85), reflecting partial regression of behavioral gains.

Regarding adaptive functioning, ABS-Arabic scores improved sharply from 2008 (*Mdn* = 75) to 2010 (*Mdn* = 225; *Z* = − 7.06, *p* <.001, *r* =.87) and continued to improve up to 2013 (*Mdn* = 261; *Z* = − 5.83, *p* <.001, *r* =.72). Between 2013 and 2019, a statistically significant attenuation of earlier gains with a large effect size was observed (*Z* = − 6.01, *p* <.001, *r* =.74), yet 2019 levels (*Mdn* = 218) remained significantly higher than pre-program scores (*p* <.001).

Together, these results demonstrate significant and durable effects of the FC-EIBI program on autism severity, maladaptive behaviors, and adaptive functioning over 11 years (Table 1).

### Exploratory Analyses by Age at Treatment Onset

Exploratory analyses examined whether long-term developmental trajectories differed by age at initiation of early intensive behavioral intervention (EIBI; 1–3 years, 3–5 years, ≥ 5 years). Repeated-measures analyses including time (2008, 2010, 2013, 2019) and age-at-onset group revealed a robust main effect of time across all outcome measures, indicating substantial improvement following intervention with partial attenuation over time.

For autism symptom severity (CARS), a modest Time × Age interaction emerged after correction for sphericity (See Supplementary Material Figure S1 and Table S1a). Greenhouse–Geisser *p =*.044, partial *η² =* 0.072. Visual inspection of trajectories suggested broadly similar patterns of improvement and partial symptom re-emergence across age groups, with no clear or clinically distinct separation between groups.

For maladaptive behaviors (ABC), the Time × Age interaction was not statistically significant (Greenhouse–Geisser *p =*.214), indicating comparable longitudinal trajectories across age-at-onset groups (Supplementary Material Figure S2 and Table S1b).

Adaptive functioning (ABS) demonstrated a pronounced Time × Age interaction (see Figure S3 and Table S1c in the Supplementary Material). Greenhouse–Geisser *p <*.001, partial *η² =* 0.236. Children who initiated EIBI earlier showed steeper gains in adaptive functioning and greater long-term maintenance relative to those who began intervention later. Given the exploratory nature of these analyses and the absence of a comparison group, these findings should be interpreted as descriptive and hypothesis-generating rather than as evidence of causal age-dependent effects. 

### ABS-Arabic Subdomains Outcomes

Changes across the six ABS-Arabic subdomains—Social Interaction, Communication and Language, Self-Care, Motor Skills, Cognitive/Basic Knowledge, and Personal–Emotional Adaptation—were examined using Friedman tests for non-parametric repeated measures (Table 2). All omnibus tests were statistically significant, indicating robust changes across the four assessment points (2008, 2010, 2013, 2019), *χ²*(3, *N* = 66) ranged from 139.79 to 178.19, all *p*s < 0.001; Kendall’s *W* = 0.71–0.90, representing extensive to substantial effects according to Cohen’s (1988) benchmarks. Participants demonstrated substantial improvements from pre- to post-test (2008–2010) across all subdomains. Wilcoxon signed-rank analyses, adjusted using Bonferroni correction (α = 0.0083), revealed large effect sizes (*r* =.86–0.88) for all early comparisons.

**Table 2:** Descriptive statistics and Friedman test results for ABS-Arabic subdomains (2008–2019)

Subdomain	2008 M (SD)	2010 M (SD)	2013 M (SD)	2019 M (SD)	*χ² (3,N = 66)*	*p*	Kendall’s W
Social Interaction	7.45 (6.50)	42.27 (9.78)	29.26 (13.29)	28.06 (13.58)	178.19	< 0.001	0.90
Communication & Language	7.38 (7.62)	41.77 (9.36)	36.97 (15.17)	35.12 (16.14)	162.37	< 0.001	0.82
Self-Care	16.20 (12.69)	46.29 (10.00)	42.11 (15.69)	41.71 (16.15)	147.63	< 0.001	0.75
Motor Skills	22.62 (12.36)	49.30 (11.37)	46.47 (13.26)	46.02 (14.08)	143.46	< 0.001	0.73
Cognitive/Basic Knowledge	13.33 (6.41)	44.32 (10.02)	40.30 (17.19)	39.08 (18.01)	139.79	< 0.001	0.71
Personal–Emotional Adaptation	8.64 (8.93)	38.89 (8.45)	32.15 (12.78)	30.52 (11.98)	162.18	< 0.001	0.82

*M* = mean; *SD* = standard deviation; χ² = chi-square value from Friedman test; *W* = Kendall’s coefficient of concordance (effect size). All Friedman tests were significant at *p* <.001. Kendall’s *W* values of 0.71–0.90 represent large to very-large effects

For instance, Social Interaction, *Z* = − 7.09, *p* <.001, *r* =.87, and Communication and Language, *Z* = − 7.08, *p* <.001, *r* =.87, showed the strongest immediate gains, while Self-Care (*Z* = − 7.11, *p* <.001, *r* =.87) and Motor Skills (*Z* = − 7.02, *p* <.001, *r* =.86) displayed comparable improvements (Table 3).

**Table 3:** Bonferroni-adjusted Wilcoxon signed-rank pairwise comparisons for ABS-Arabic subdomains (*N* = 66)

Subdomain	Comparison (Year 1 – Year 2)	Z	*p* (2-tailed)	*r*	Direction (↑ = improvement, ↓ = regression)
Social Interaction	2010–2008	–7.09	< 0.001	0.87	↑
	2013–2008	–7.06	< 0.001	0.87	↑
	2019–2008	–7.06	< 0.001	0.87	↑
	2013–2010	–6.99	< 0.001	0.86	↓
	2019–2010	–6.94	< 0.001	0.85	↓
	2019–2013	–3.80	< 0.001	0.47	↓
Communication & Language	2010–2008	–7.08	< 0.001	0.87	↑
	2013–2008	–7.07	< 0.001	0.87	↑
	2019–2008	–7.07	< 0.001	0.87	↑
	2013–2010	–4.69	< 0.001	0.58	↓
	2019–2010	–5.03	< 0.001	0.62	↓
	2019–2013	–5.41	< 0.001	0.67	↓
Self-Care	2010–2008	–7.11	< 0.001	0.87	↑
	2013–2008	–7.08	< 0.001	0.87	↑
	2019–2008	–7.07	< 0.001	0.87	↑
	2013–2010	–4.06	< 0.001	0.50	↓
	2019–2010	–3.96	< 0.001	0.49	↓
	2019–2013	–2.58	0.010	0.32	↓
Motor Skills	2010–2008	–7.02	< 0.001	0.86	↑
	2013–2008	–7.01	< 0.001	0.86	↑
	2019–2008	–7.01	< 0.001	0.86	↑
	2013–2010	–4.78	< 0.001	0.59	↓
	2019–2010	–4.55	< 0.001	0.56	↓
	2019–2013	–2.66	0.008	0.33	↓
Cognitive/Basic Knowledge	2010–2008	–7.07	< 0.001	0.87	↑
	2013–2008	–7.06	< 0.001	0.87	↑
	2019–2008	–7.05	< 0.001	0.87	↑
	2013–2010	–3.49	< 0.001	0.43	↓
	2019–2010	–3.66	< 0.001	0.45	↓
	2019–2013	–4.40	< 0.001	0.54	↓
Personal–Emotional Adaptation	2010–2008	–7.13	< 0.001	0.88	↑
	2013–2008	–7.01	< 0.001	0.86	↑
	2019–2008	–6.98	< 0.001	0.86	↑
	2013–2010	–5.43	< 0.001	0.67	↓
	2019–2010	–5.94	< 0.001	0.73	↓
	2019–2013	–5.21	< 0.001	0.64	↓

*Wilcoxon signed-rank tests adjusted with Bonferroni correction (α = 0.05/6 = 0.0083).*

*Z = standardized Wilcoxon test statistic; p = two-tailed probability; r = effect size calculated as |Z|/√N (√66 ≈ 8.12); ↑ = significant improvement; ↓ = significant regression; ns = non-significant after correction. Effect-size interpretation: small (0.10)*,* medium (0.30)*,* large (0.50)*

Between the post-test (2010) and the first follow-up (2013), significant regressions emerged across all subdomains, though effect sizes varied. The largest decline in functioning occurred in Personal–Emotional Adaptation (*Z* = − 5.43, *p* <.001, *r* =.67) and Communication and Language (*Z* = − 4.69, *p* <.001, *r* =.58), whereas Self-Care (*Z* = − 4.06, *p* <.001, *r* =.50) and Motor Skills (*Z* = − 4.78, *p* <.001, *r* =.59) showed more large declines (Table 3).

Further decline in functioning between 2013 and 2019 were evident, particularly for Communication and Language (*Z* = − 5.41, *p* <.001, *r* =.67) and Personal–Emotional Adaptation (*Z* = − 5.21, *p* <.001, *r* =.64). Medium regressions were noted for Self-Care (*Z* = − 2.58, *p* =.010, *r* =.32, ns after correction) and Motor Skills (*Z* = − 2.66, *p* =.008, *r* =.33). Despite these declines, 2019 scores remained significantly higher than pre-intervention levels across all subdomains (all *Z*s ≤ − 6.98, all *p*s < 0.001, *r*s ≥ 0.85), demonstrating enduring though partially attenuated adaptive-behavior gains nearly a decade after the FC-EIBI program (Table 3).

### Δ Change during the FC-EIBI Intervention

Friedman tests revealed significant differences in the magnitude of change across the six ABS-Arabic subdomains for all study phases (Table 4). During the intervention phase (Δ₁ = 2008–2010), *χ²*(5, *N* = 66) = 88.09, *p* <.001, Kendall’s *W* = 0.27, a medium effect, indicating that improvement varied across subdomains. Bonferroni-adjusted Wilcoxon tests showed that gains were significantly greater in *Communication* and *Social Interaction* than in *Self-Care*, *Basic Knowledge*, and *Motor Skills* (e.g., *Z* = − 6.71, *p* <.001, *r* =.83).

**Table 4:** Friedman tests of change across ABS-Arabic subdomains for the three study phases (Δ₁ = 2008–2010, Δ₂ = 2010–2013, Δ₃ = 2013–2019)

Phase	*χ² (5,>N = 66)*	*p*	Kendall’s W	Main subdomain pattern	Representative Wilcoxon comparisons	Z	*p*	*r*
**Δ₁ (2008–2010)**	88.09	< 0.001	0.27	Communication ≈ Social > Self-Care ≈ Basic > Motor ≈ Emotional	Comm vs. Motor (↑)	−6.71	< 0.001	0.83
					Social vs. Basic (↑)	−4.59	< 0.001	0.57
**Δ₂ (2010–2013)**	116.80	< 0.001	0.35	Social > Emotional > Basic ≈ Self-Care > Motor	Social vs. Motor (↓)	−7.02	< 0.001	0.87
					Emotional vs. Self-Care (↓)	−5.86	< 0.001	0.72
**Δ₃ (2013–2019)**	23.89	< 0.001	0.07	Communication ≈ Emotional > Self-Care ≈ Motor	Comm vs. Motor (↓)	−4.37	< 0.001	0.54
					Emot vs. Motor (↓)	−2.85	0.004	0.35

Δ₁ = change during the FC-EIBI intervention; Δ₂ = early follow-up; Δ₃ = long-term follow-up. χ² = Friedman chi-square statistic; p = asymptotic significance (two-tailed); W = Kendall’s coefficient of concordance (effect size); Z = standardized Wilcoxon test statistic; r = effect size computed as |Z|/√N (√66 ≈ 8.12). Bonferroni-adjusted α = 0.05/15 = 0.0033. ↑ = significant improvement; ↓ = significant regression. According to Cohen (1988),r ≥.50 = large,0.30 = medium, 0.10 = small

In the early follow-up (Δ₂ = 2010–2013), changes again differed significantly, *χ²*(5, *N* = 66) = 116.80, *p* <.001, *W* = 0.35. Regression was greatest in *Social Interaction* (*Z* = − 7.02, *p* <.001, *r* =.87) and *Emotional Adaptation* (*Z* = − 5.86, *p* <.001, *r* =.72), whereas *Motor Skills* showed minimal decline.

During the long-term follow-up (Δ₃ = 2013–2019), differences across subdomains were more minor but still significant, *χ²* (5, *N* = 66) = 23.89, *p* <.001, *W* = 0.07. Communication and Emotional Adaptation declined more than Self-Care and Motor (*Z* = − 4.37, *p* <.001, *r* =.54). Median change values indicated clinically meaningful improvement: participants’ median CARS scores decreased by 5 points, ABC totals by 35 points, and ABS-Arabic totals increased by 186 points from baseline to 2013, with modest declines (ΔABS = − 43) from 2013 to 2019.

Overall, the pattern indicates that social–communicative and emotional domains were the most responsive to early intervention but also the most vulnerable to later partial decline in adaptive functioning. In contrast, practical daily living and motor skills were comparatively stable. This pattern of selective maintenance versus decline suggests that the durability of early behavioral gains may depend on the nature of the skill and its opportunity for continued use.

## Discussion

This 8 years longitudinal follow-up of the Future Center’s Early Intensive Behavioral Intervention (FC-EIBI) provides rare evidence that early behavioral gains can persist under conditions of profound sociopolitical instability. Across four waves spanning 11 years, participants demonstrated significant improvements in autism symptom severity (CARS), reductions in maladaptive behaviors (ABC), and improvements in adaptive functioning (ABS-Arabic) from baseline to post-treatment, with benefits maintained at both follow-ups. The only attenuation observed between 2013 and 2019 was confined to social-communication facets, whereas self-care and motor domains remained stable. These findings indicate that core competencies acquired during intensive early intervention generalized and consolidated over time, with only modest erosion in areas most dependent on sustained social opportunity. These domain-specific patterns empirically support Fossum et al.’s (2025) longitudinal framework, Specifically, our findings exemplify the proposed interaction between skill type and environmental continuity -where maintenance hinges on the availability of real-life practice contexts- in which the maintenance of adaptive gains depends less on initial IQ and more on the continuity of skill practice and environmental scaffolding. The preserved self-care and motor skills illustrate this ‘practice continuity’ mechanism, whereas the decline in social communication reflects reduced opportunities for reciprocal interaction. Interpreted developmentally, these findings suggest that the consolidation of adaptive functioning relies on environmental practice opportunities rather than on cognitive maturation alone.

In exploratory analyses, we also examined whether long-term developmental trajectories differed by age at EIBI initiation. Across outcome domains, all age-at-onset groups demonstrated substantial improvement following intervention and maintenance of gains at long-term follow-up. For autism symptom severity and maladaptive behaviors, trajectories were broadly comparable across age groups, suggesting that long-term patterns of change were not strongly differentiated by age at treatment onset. In contrast, adaptive functioning showed a more pronounced age-related pattern, with earlier initiation associated with steeper gains and greater long-term maintenance. Importantly, these findings are exploratory and descriptive in nature and should not be interpreted as evidence of causal age-dependent effects. Rather, they highlight potential heterogeneity in long-term adaptation that warrants further investigation in adequately powered and controlled studies. 

The durability of outcomes observed here aligns with prior longitudinal reports from higher-resource settings showing that EIBI-related gains can be maintained for many years (e.g., Estes et al., 2015; Smith et al., 2021) and extends that literature by demonstrating persistence in a conflict-affected, low-resource context. From a developmental perspective, the pattern closely accords with longitudinal models that foreground adaptive functioning as a malleable, context-sensitive endpoint (Bal et al., 2021; Szatmari et al., 2019). Fossum et al. (2025) emphasized that early adaptive skills predict emerging-adult competence more strongly than IQ when the former is modeled directly, and that continuity of practice and participation shapes trajectories through adolescence. The present cohort’s stability in self-care and motor domains—skills that lend themselves to home-based rehearsal—contrasts with declines in social-communication domains, which plausibly depend on consistent peer interaction and structured schooling. This divergence fits Fossum and colleagues’ argument that adaptive behavior is sustained when environments afford regular opportunities to deploy learned skills.

The Syrian context renders this sustained improvement especially noteworthy. Since 2011, families have faced school closures, displacement, and fragmented access to clinical services. In our earlier follow-up, we observed that the frequency of structured learning trials remained strongly associated with outcomes (Mounzer et al., 2023), suggesting that parent-mediated practice helped preserve gains after formal services ended. Although the current study did not re-measure intensity or parental engagement in 2019, the domain-specific pattern—stability in daily-living skills versus declines in social communication—indirectly points to environmental constraints rather than a loss of capacity per se. Put differently, where families could feasibly continue to embed routines (hygiene, dressing, household tasks), gains were maintained; where prolonged social deprivation limited conversational and peer practice, a modest partial decline in adaptive functioning emerged. This interpretation is consistent with developmental-ecological accounts in which adaptive functioning reflects an ongoing match between individual competencies and environmental affordances.

Clinically, three implications follow. First, in low-resource or disrupted systems, front-loading high-quality early intervention that explicitly targets generalization to home routines may yield benefits that are more resilient to later service gaps. Second, to protect socially contingent skills, programs should include low-cost maintenance strategies feasible under instability (e.g., parent-led conversation games, peer-buddy phone sessions, scripted play at home), alongside periodic tele-coaching when in-person services are unavailable. Third, outcome monitoring in such settings should prioritize adaptive functioning—not only symptom scales—because it better captures everyday competence and aligns with life-course models (Fossum et al., 2025).

This study also addresses equity and cultural generalizability gaps in the intervention literature. Much EIBI evidence has accrued in WEIRD contexts (Muthukrishna et al., 2020), where service continuity is often taken for granted. Demonstrating long-term maintenance in a conflict-affected, Arabic-speaking population strengthens the ecological validity of ABA-based approaches. It also underscores the role of cultural adaptation (training paraprofessionals, parent coaching in the home language, context-appropriate materials). The Future Center model’s combination of intensive center-based instruction with structured parent involvement may be particularly suited to systems where professional capacity is limited.

Strengths of this study include the long observation window, exceptional retention (98.5%), consistent use of standardized Arabic measures across all waves, and fidelity monitoring for both intervention (2008–2010) and the 2019 assessments. Replicating measurements across waves permits a conservative test of maintenance. Analytically, nonparametric procedures were justified by departures from normality, and effect sizes were reported consistently, supporting interpretability.

Several limitations should be noted. First, the sample predominantly consisted of individuals with low intellectual functioning, reflecting the population most commonly offered EIBI in the study context. Consequently, the present findings should not be generalized to autistic individuals with average or above-average cognitive abilities, for whom developmental trajectories and responsiveness to early intervention may differ. Second, the absence of a comparison group and the lack of statistical control for baseline IQ, autism severity, or demographic variables preclude causal inferences regarding intervention effects. Although the observed pattern is consistent with intervention-related change, natural maturation and secular influences cannot be fully excluded. This limitation reflects the ethical and contextual constraints of conducting long-term research in conflict-affected, low-resource settings; future studies should integrate comparison cohorts or analytically model baseline severity to better isolate intervention-specific contributions. Third, we did not collect 2019 measures of intervention exposure, parental engagement, or schooling continuity, which precluded formal tests of longitudinal predictors of maintenance. Future waves should integrate these indices to evaluate moderators and mediators of sustained outcomes (cf. Fossum et al., 2025). Fourth, adaptive behavior was assessed using caregiver report and clinician ratings. While these instruments demonstrate strong psychometric properties and cultural relevance among Arabic-speaking populations, the inclusion of multi-informant data (e.g., teacher reports or direct observation) would further strengthen validity where feasible. Moreover, although the instruments were administered longitudinally with acceptable reliability, formal tests of cross-cultural measurement invariance (e.g., configural, metric, or scalar invariance) were not conducted. Accordingly, results should be interpreted as reflecting within-individual change over time rather than equivalence to normative scores derived from other cultural contexts. Finally, the modest attenuation observed in social-communication domains warrants careful monitoring. Future research should experimentally evaluate low-cost maintenance strategies—such as parent-mediated conversation practice, peer-buddy routines, and tele-coaching—as pragmatic approaches to preserving socially contingent skills during service disruptions. Although detailed race and ethnicity data were not collected, the sample represents Arabic-speaking families within a specific sociocultural and geopolitical context. Findings should therefore be interpreted with consideration of cultural, linguistic, and contextual factors, and generalization beyond similar settings should be made with caution.

In conclusion, this longitudinal study shows that early intensive behavioral intervention can yield durable improvements in everyday functioning that persist for 8 years even amid sociopolitical disruption. The domain-specific pattern—stability in routinized daily living and motor skills with declines in socially contingent abilities—highlights the importance of ongoing opportunities for practice. Together, these findings highlight that sustaining treatment effects requires both effective early intervention and socio-ecological stability that allows continued skill use. By placing adaptive functioning at the center of outcome evaluation and interpreting change through a developmental-ecological lens, these findings connect early intervention to life-course competence and offer concrete directions for sustaining gains in low-resource and conflict-affected settings worldwide Friedman tests of change across ABS-Arabic subdomains for the three study phases (Δ₁ = 2008–2010, Δ₂ = 2010–2013, Δ₃ = 2013–2019).

## Supplementary Information

Below is the link to the electronic supplementary material.

Supplementary Material 1 (DOCX 476 KB)

## Data Availability

The datasets generated and analyzed during the 2019 follow-up are not publicly available due to ethical restrictions and data protection policies concerning minors with ASD. However, de-identified data may be obtained from the corresponding author upon reasonable request, provided appropriate ethical clearance has been obtained.
