# Supplementary material for: Sustained Autism Outcomes Eight Years After Early Intensive Behavioral Intervention in a Conflict-Affected Low-Resource Setting: A Longitudinal Follow-Up Study
Source: Res Child Adolesc Psychopathol. 2026 Feb 26;54(2):39. doi: 10.1007/s10802-026-01438-x (PMC12945914; doi:10.1007/s10802-026-01438-x)
Supplement: Supplementary file 1 — Supplementary Material 1 (DOCX 476 KB) [file 10802_2026_1438_MOESM1_ESM.docx]

# Supplementary Material

## Figures

**Figure S1***CARS trajectories by age at treatment onset across assessment waves.*
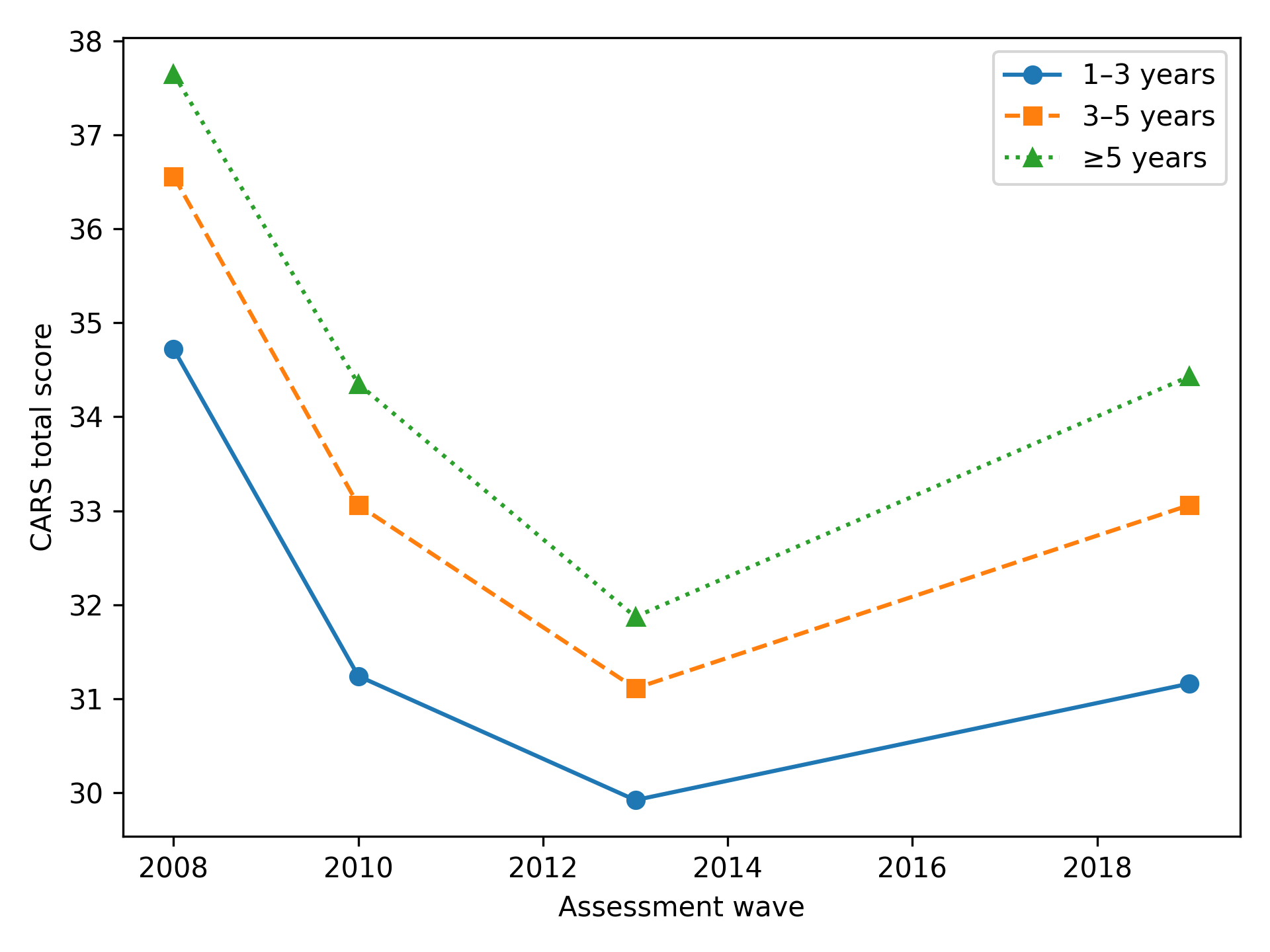


Note. Values represent means across assessment waves. Lower scores indicate less severe autism symptoms.

**Figure S2***ABC trajectories by age at treatment onset across assessment waves.*
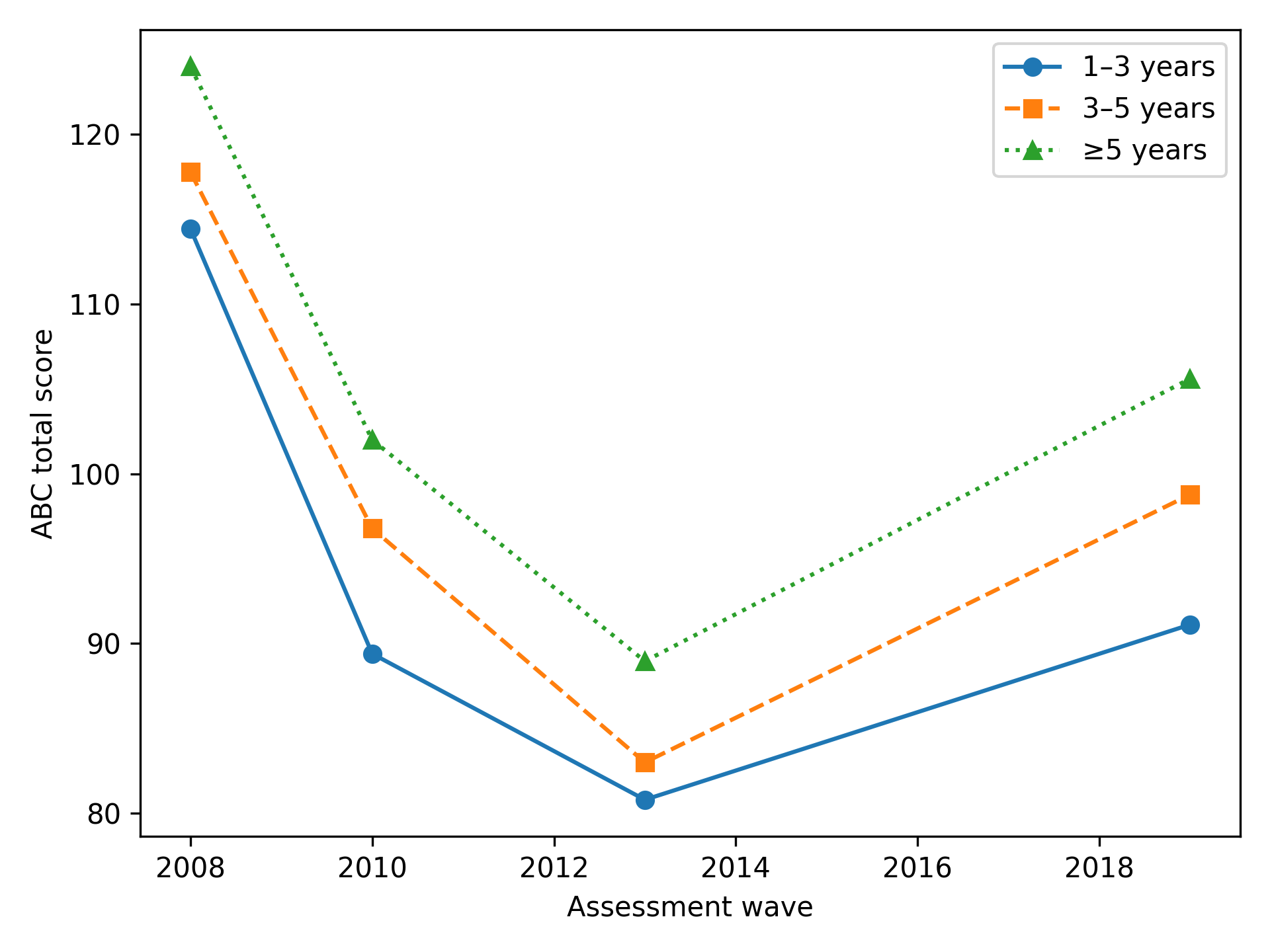


Note. Values represent means across assessment waves. Lower scores indicate fewer maladaptive behaviors.

Figure S3
ABS–Arabic trajectories by age at treatment onset across assessment waves.


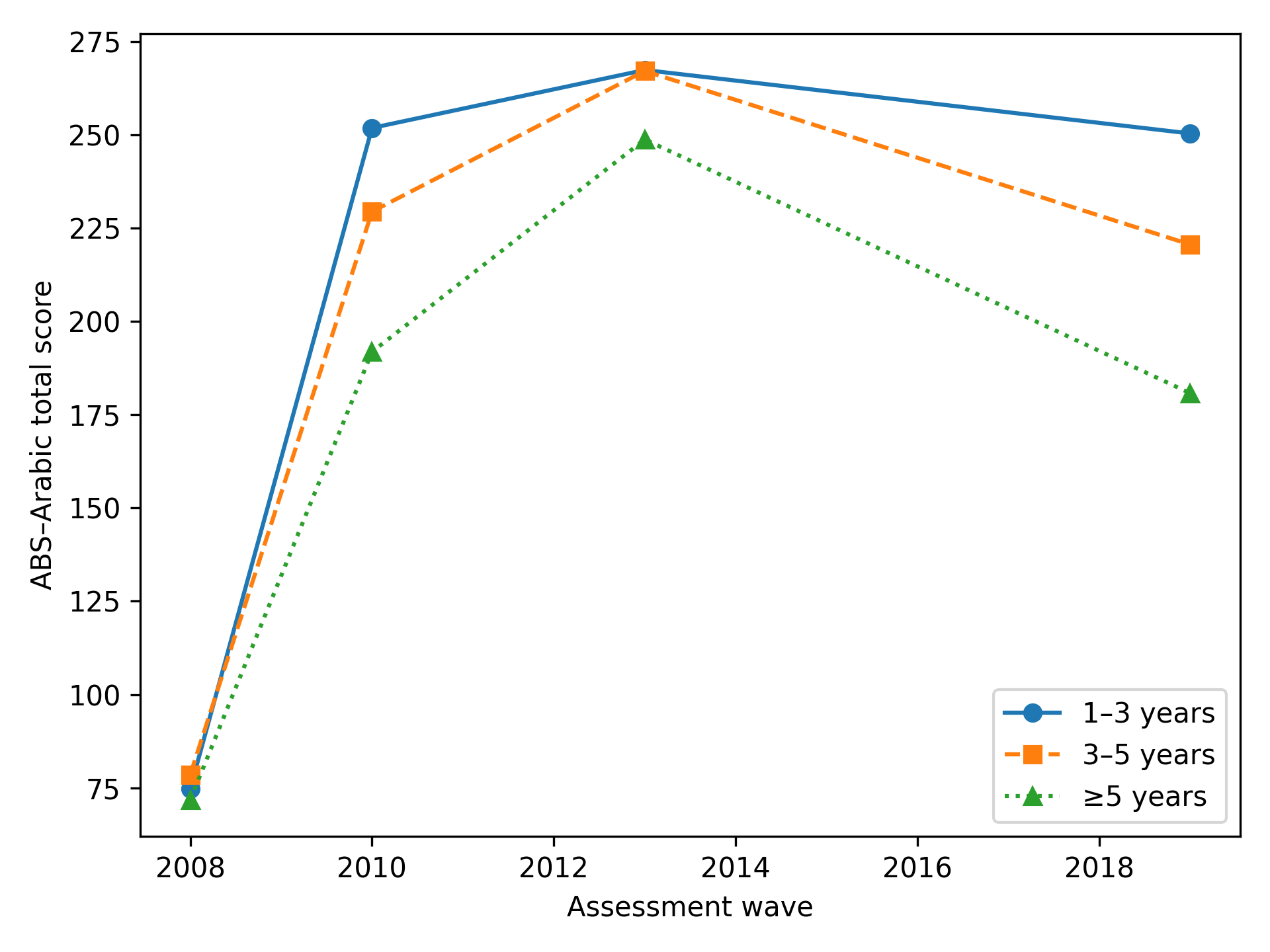


## Tables

**Table S1***Descriptive statistics by age at treatment onset and assessment wave.*

### Table S1a

CARS (Childhood Autism Rating Scale; lower scores indicate less severe autism symptoms)

| Age at EIBI onset | 2008 | 2010 | 2013 | 2019 |
| --- | --- | --- | --- | --- |
| 1–3 years | 34.72 (3.38) | 31.24 (3.82) | 29.92 (4.11) | 31.16 (3.71) |
| 3–5 years | 36.56 (4.66) | 33.06 (5.34) | 31.11 (5.35) | 33.06 (5.61) |
| ≥5 years | 37.65 (6.09) | 34.35 (7.22) | 31.87 (7.12) | 34.43 (7.15) |
| Total | 36.24 (4.91) | 32.82 (5.68) | 30.92 (5.62) | 32.82 (5.71) |

Note. Values are presented as M (SD). Age groups are defined by age at initiation of early intensive behavioral intervention (EIBI).

### Table S1b

ABC (Autism Behavior Checklist; lower scores indicate fewer maladaptive behaviors)

| Age at EIBI onset | 2008 | 2010 | 2013 | 2019 |
| --- | --- | --- | --- | --- |
| 1–3 years | 114.44 (26.52) | 89.40 (22.78) | 80.80 (21.66) | 91.12 (24.08) |
| 3–5 years | 117.78 (28.78) | 96.78 (31.33) | 83.00 (27.49) | 98.78 (33.55) |
| ≥5 years | 124.00 (32.36) | 102.00 (34.15) | 88.96 (32.77) | 105.61 (34.93) |
| Total | 118.68 (29.12) | 95.80 (29.54) | 84.24 (27.34) | 98.26 (30.99) |

Note. Values are presented as M (SD). Age groups are defined by age at initiation of early intensive behavioral intervention (EIBI).

### Table S1c

ABS–Arabic (Adaptive Behavior Scale; higher scores indicate stronger adaptive abilities)

| Age at EIBI onset | 2008 | 2010 | 2013 | 2019 |
| --- | --- | --- | --- | --- |
| 1–3 years | 74.76 (37.52) | 251.80 (68.28) | 267.32 (51.78) | 250.32 (71.91) |
| 3–5 years | 78.50 (60.25) | 229.39 (76.46) | 267.06 (54.06) | 220.56 (75.49) |
| ≥5 years | 71.78 (52.32) | 191.83 (92.30) | 248.70 (58.81) | 180.74 (93.07) |
| Total | 74.74 (49.02) | 224.79 (82.49) | 260.76 (54.81) | 217.95 (85.01) |

Note. Values are presented as M (SD). Age groups are defined by age at initiation of early intensive behavioral intervention (EIBI).
